# Primary Osteosarcoma of the Breast in a Patient Treated Previously for Invasive Ductal Carcinoma: An Unusual Presentation of a Very Rare Primary Breast Malignancy

**DOI:** 10.1155/2020/1594127

**Published:** 2020-01-16

**Authors:** David Malcolm Milne, Navin Sookar, Srikanth Umakanthan, Fidel Rampersad, Lyronne Olivier, Jameel Ali

**Affiliations:** ^1^General Surgery Department, General Hospital Port of Spain, Trinidad and Tobago; ^2^Department of Women's Health, St. James Medical Complex, Trinidad and Tobago; ^3^Faculty of Medical Sciences, Department of Paraclinical Sciences, The University of the West Indies, Trinidad and Tobago; ^4^Radiology Department, Eric Williams Medical Sciences Complex, Trinidad and Tobago; ^5^General Surgery Department, Sangre Grande Regional Hospital, Trinidad and Tobago

## Abstract

Primary osteogenic sarcoma of the breast is a rare clinical entity with few cases described in the literature. Unfortunately, the prognosis for these patients is poor when compared to invasive carcinomas of the breast. We report a case of a 58-year-old female who developed a primary osteogenic sarcoma of the breast five years after being treated for invasive carcinoma of the ipsilateral breast without the use of radiotherapy.

## 1. Introduction

Breast lumps are the most common presenting complaint (40%) among patients presenting to general practice clinics with breast symptoms [1]. Among those referred to a tertiary centre, breast cancer is found to be the cause of the breast lump in 36% of patients [2].

Breast cancer is the most prevalent form of cancer observed in females globally, with 2.1 million persons affected each year. It accounts for the most cancer-related deaths among women with 627,000 dying from the disease in 2018 [3].

Carcinomas account for the majority of breast cancers with the contribution of sarcomas being less than 1% [4, 5]. Primary osteogenic sarcoma of the breast is particularly rare with published data being limited to case reports and small series [6].

When compared to breast carcinoma, primary osteogenic sarcoma has a sombre 5-year survival of 38% [6]. Given the rarity of this tumour and the reported poor outcomes of affected patients, it is critical that cases are reported in the literature to expand the available knowledge base in the hopes of eventually improving patient care.

We report the case of a patient who developed a primary osteogenic sarcoma of the breast five years after being treated for invasive carcinoma of the ipsilateral breast.

## 2. Case Report

A 58-year-old female presented with a 3 cm mass in the lower inner quadrant of the left breast. Ultrasound and mammography revealed a Breast Imaging Reporting and Data System [7] (BI-RADS) 4 lesion. Core biopsy of the lesion showed a grade 2 invasive ductal adenocarcinoma. The patient had wide local excision of the lesion along with axillary lymph node dissection.

Pathological examination revealed a T2 N0 M0 grade 1 invasive ductal carcinoma with 14 examined lymph nodes showing no evidence of metastases. The patient subsequently refused adjuvant radiation and chemotherapy.

Five years after her surgery, the patient presented with a painless, mobile, 15 cm mass involving the upper and lower outer quadrants of the left breast. No evidence of nipple retraction or discharge was observed. Mammography revealed a BI-RADS 5 lesion. A core biopsy showed breast tissue containing areas of bone formation with partially calcified osteoid material surrounded by stellate and spindle-shaped stromal cells. There was also osteoclast-like giant cells present.

Contrast-enhanced computed tomography (CT) of the chest and abdomen revealed a large lobulated left breast mass with cystic and calcific foci, measuring 7.1 × 10.7 × 11.6 cm with no evidence of invasion into the chest wall or skin. No pulmonary, hepatic, or bony lesions were identified.

**Figure 1:**
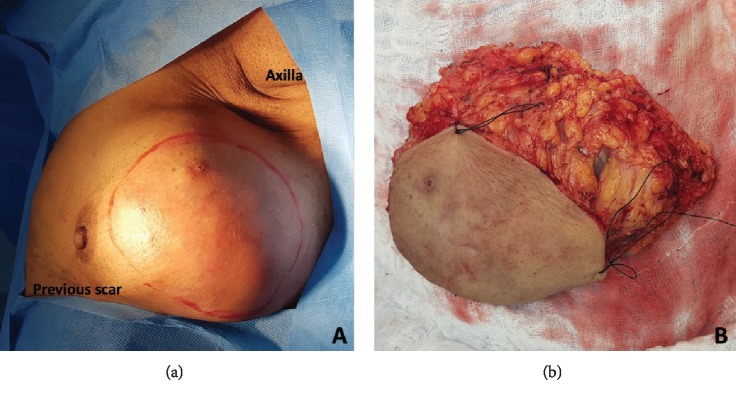
(a) Preoperative marking of the breast mass. (b) Surgical specimen after wide local excision.

The patient refused mastectomy and chose to have a wide local excision of the breast mass (see Figure 1).

**Figure 2:**
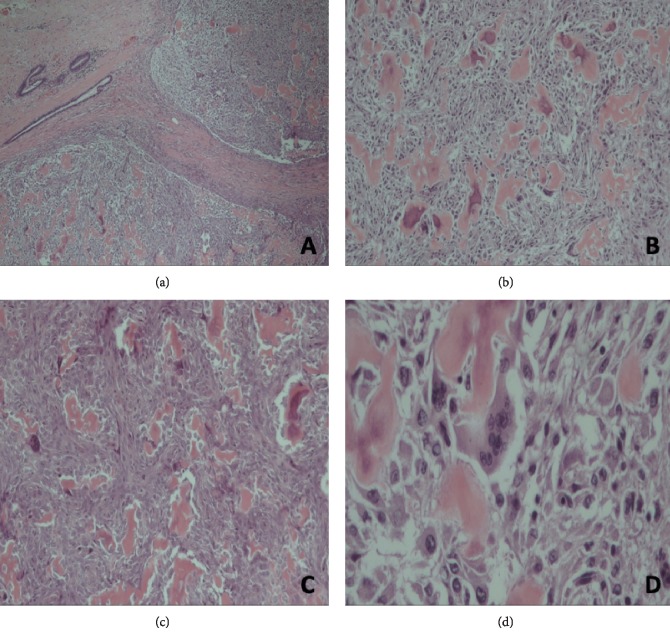
(a) Malignant tumour with surrounding breast parenchyma (H&E, ×4). (b) Malignant tumour composed of neoplastic woven bone intimately admixed with pleomorphic tumour cells (H&E, ×10). (c) Neoplastic woven bone exhibiting eosinophilic osteoid along with high pleomorphic tumour cells (H&E, ×10). (d) Pleomorphic tumour cells having vesicular chromatin and conspicuous nucleoli showing both osteoblastic and osteoclastic (giant cell) subtypes (H&E, ×40).

Pathologic examination of the mass showed osteogenic sarcoma with malignant cells admixed with neoplastic woven bone and frequent mitotic figures (see Figure 2). No evidence of infiltrating ductal carcinoma or ductal carcinoma in situ was observed. The tumour was noted to involve all margins. A completion mastectomy was subsequently performed, which showed no evidence of residual disease on pathological examination.

A technetium-99 methylene diphosphonate bone scan was undertaken, and no evidence of primary osteosarcoma arising from bone was detected, indicating that the breast lesion was primary osteosarcoma.

## 3. Discussion

Extraskeletal osteogenic sarcoma is a rare subtype of sarcoma accounting for 0.01% of all soft tissue sarcomas [8]. These tumours typically arise from the soft tissues of the lower extremity. However, they have also been documented to arise in the head, neck, upper extremity, abdomen, and rarely from the breast [9].

Primary osteogenic sarcoma of the breast (POSB) typically presents as a painless mass with no attendant evidence of nipple discharge or retraction [10]. Similar to phyllodes tumours, POSB exhibit rapid growth which may account for the large average size (4.6 cm) at presentation [6, 11]. In contrast to skeletal osteosarcomas which tend to present at a younger age, the average age of presentation of POSB is 64 [6].

Of note, prior radiation and trauma to the affected area have been described as being present in 10% and 15% of patients, respectively, with extraskeletal osteogenic sarcomas [9, 12]. As it relates to potential risk factors for POSB, 3 cases have been reported of patients developing POSB after having radiotherapy for breast cancer [6, 13, 14]. In our case, the patient developed her tumour after having surgery for breast cancer but without having radiotherapy. The trauma of her previous surgery may have played a role in the pathogenesis of the tumour, or it is possible that previous breast cancer in and of itself is a risk factor for developing POSB independent of radiation exposure.

Useful imaging techniques for the evaluation of patients with POSB include mammography, CT, and bone scintigraphy. Mammographic findings are similar to that of fibroadenoma and may lead to misdiagnosis [15]. The lesion typically appears as a well-defined, hyperdense mass with coarse calcifications. CT plays an important role in excluding a lesion arising from the underlying bony structures as well as identifying distant metastases [10]. Skeletal scintigraphy if done preoperatively can suggest a POSB if increased uptake is noted in the lesion. It is, however, more useful as a tool to rule out primary skeletal osteosarcoma which metastasised to the breast, as was done in this case [16].

While imaging is useful in the workup of a patient with POSB, the diagnosis cannot be made without pathological assessment. The utility of both fine needle aspirate and core needle biopsy in the preoperative workup of patients with POSB has been described in the literature [17–19]. Given that POSB often appears like a benign fibroadenoma on imaging, preoperative tissue sampling can allow for an expeditious diagnosis of malignancy, thus preventing delays in the management of this biologically aggressive disease.

Numerous tumours of the breast can produce cartilage, osteoid, and bone and hence must be included in the differential diagnosis for a case of POSB. The main differentials are metaplastic carcinoma, malignant phyllodes tumours with osteosarcomatous differentiation (or heterologous elements), and benign heterotopic ossification/fasciitis ossificans [19, 20]. These tumours, along with metastatic osteosarcoma of the bone must be ruled out before a diagnosis of POSB is made. This can be achieved by employing the diagnostic criteria outlined by Allan and Solle [8] as follows: (1) exclusion of a mixed malignant mesenchymal tumour by the presence of a consistent morphologic pattern of sarcomatous tissue, (2) the presence of malignant osteoid and/or bone, and (3) ruling out a bony origin for the tumour. The confirmation of a consistent morphologic pattern necessitates thorough sampling of the lesion. This is of particular importance in ruling out a phyllodes tumour which may exhibit stromal overgrowth, requiring extensive sectioning to identify characteristic epithelial components [21].

Given the rarity of this condition, there is a paucity of data to guide management. Similar to sarcomas arising at other locations, POSB tends to be locally aggressive tumours with a propensity to spread via the blood as opposed to the characteristic lymphatic spread observed in carcinoma of the breast [18]. As such, the primary aim of surgical management of these patients is to resect the tumour to clear margins which may be achieved via a simple mastectomy or wide local excision. However, given the large average size at presentation and a high local recurrence rate of 28%, mastectomy is likely to be the most judicious option for the majority of patients [6]. In the most extensive series of patients with POSB, 20 patients underwent axillary lymph node dissection, all of which were negative for metastases. Therefore, in the absence of clinically suspicious nodes, axillary assessment may be unnecessary [6].

Forty-one per cent of patients with POSB develop metastases which highlight the need for effective adjuvant therapy [6]. The role of chemotherapy in the management of patients with POSB is uncertain with limited reports in the literature with differing regimens and outcomes [22–26]. However, because of the dramatic improvement in survival from chemotherapy administration for patients with primary osteosarcoma of the bone, it is reasonable to consider its use for a patient with POSB until better evidence is available to guide management [22]. Based on limited published works, radiotherapy does not appear to improve outcomes [27].

## 4. Conclusion

Primary osteogenic sarcoma of the breast is a rare tumour which can mimic a carcinoma on clinical presentation. Thorough pathological examination, as well as investigations to rule out a bony primary, is necessary before a diagnosis is made. Given the limited available data to guide management, further research is needed to optimise the treatment of this aggressive disease.
